# Complex Hand Polydactyly: A Case Report and Literature Review

**DOI:** 10.7759/cureus.20856

**Published:** 2021-12-31

**Authors:** Israa Alzarmah, Tanveer A Bhat, Eyad Nawwab

**Affiliations:** 1 Plastic, Reconstructive and Aesthetic Surgery, Royal College of Surgeons in Ireland, Dublin, IRL; 2 Department of Plastic and Reconstructive Surgery, King Saud Medical City, Riyadh, SAU

**Keywords:** congenital malformation, congenital anomaly, supernumerary digit, duplication, hyperdactyly, polydactyly

## Abstract

Polydactyly is a common congenital malformation in which extra digits are present in at least one extremity. It has various presentations, and it can be an isolated anomaly or part of other diseases. Most isolated polydactyly cases are sporadic and unilateral, but there is an increased incidence in some populations. Polydactyly is multifactorial and can occur in different forms. Its main line of treatment is surgery to improve cosmesis and functioning. In this report, we present a rare case of bilateral complex hand polydactyly in a one-year-seven-months old girl of African descent. She is otherwise healthy with no family history of malformations. The pattern is not consistent with any syndromic disease. She subsequently underwent surgical resection of the extra digits.

## Introduction

Polydactyly is a common congenital anomaly with variable morphologic phenotypes. It can present in isolation or as part of syndromic diseases [1,2]. When it occurs in isolation, it is typically inherited in an autosomal dominant fashion [3,4]. On the other hand, when it is associated with other diseases and syndromes, it tends to be autosomal recessive [3,4]. Polydactyly is a multifactorial, multigenetic disorder as many genes have been found to play a role in its pathogenesis [2,5,6]. Furthermore, polydactyly is classified based on the location of the extra digit into preaxial (radial-sided polydactyly), central, and postaxial (ulnar-sided polydactyly) [7]. Patients require full workup to rule out syndromes that may be associated with polydactyly and to fully evaluate the bones and surrounding tissues. Radiographic imaging assists in preoperative planning, but it can be inconclusive in some cases due to incomplete ossification. It is mainly crucial and useful in preaxial polydactyly [7]. Treatment should be individualized taking into consideration the type and severity of polydactyly. Polydactyly, especially the complex form, is challenging surgically given its different presentations and associations. Some patients may even require multiple surgeries to resect the extra digits and restore functioning. In our study, we present a case of a rare, complex polydactyly of the hand outlining our surgical approach and discussing polydactyly.

## Case presentation

**Figure 1:**
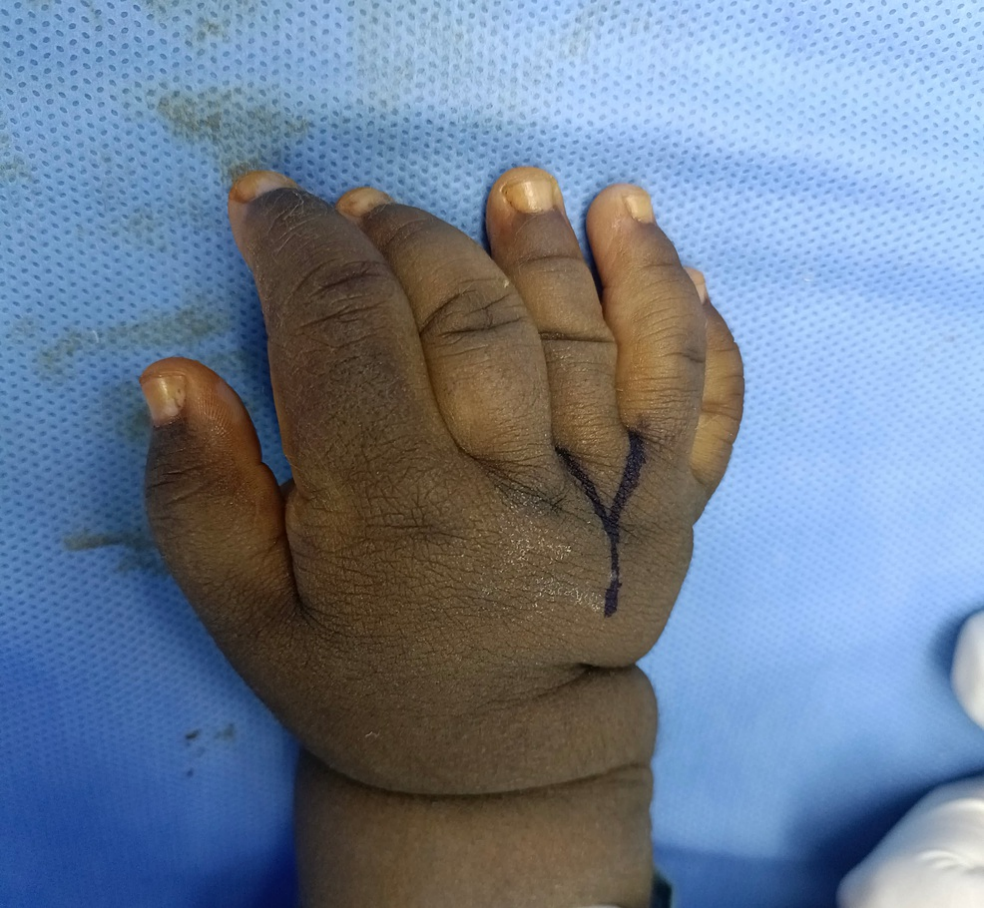
Preoperative clinical picture of the patient’s right hand showing an extra central digit and marking of the incision site on the dorsum of the hand

**Figure 2:**
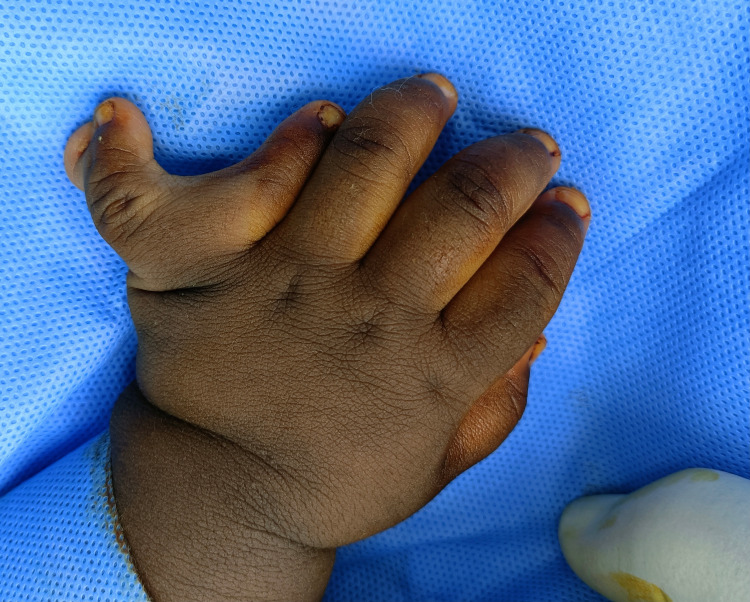
Preoperative clinical picture of the patient’s left hand showing an extra postaxial digit with bifid nail plates

A mother presented to our plastic surgery clinic with her one-year-seven-months old girl complaining of duplication of fingers of both hands. The toddler is otherwise healthy. No other abnormalities were detected at birth and thereafter. She is of African descent and is the product of non-consanguineous marriage and uncomplicated full-term pregnancy. She has an elder brother who is medically free with no history of polydactyly. There is no family history of similar deformities or any genetic disorders. On examination, a central extra digit was noted in the right hand (Figure 1) , and a postaxial extra digit was seen in the left hand (Figure 2).

**Figure 3:**
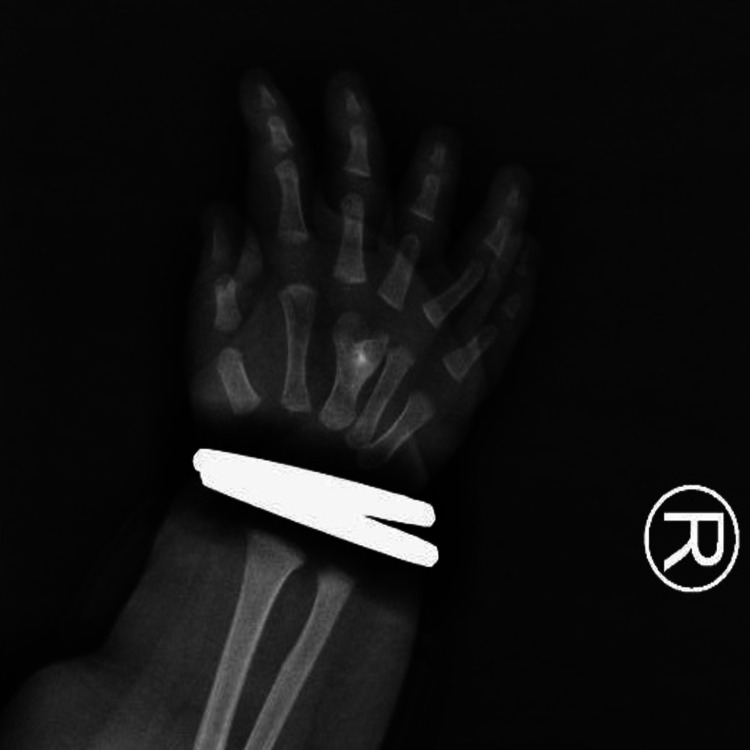
X-rays of the patient’s right hand showing an extra phalanx centrally with a bifid third metacarpal bone

No other congenital abnormalities were found. X-rays of the right hand revealed central polydactyly with a bifid third metacarpal bone involving the distal third along with the joint (Figure 3).

**Figure 4:**
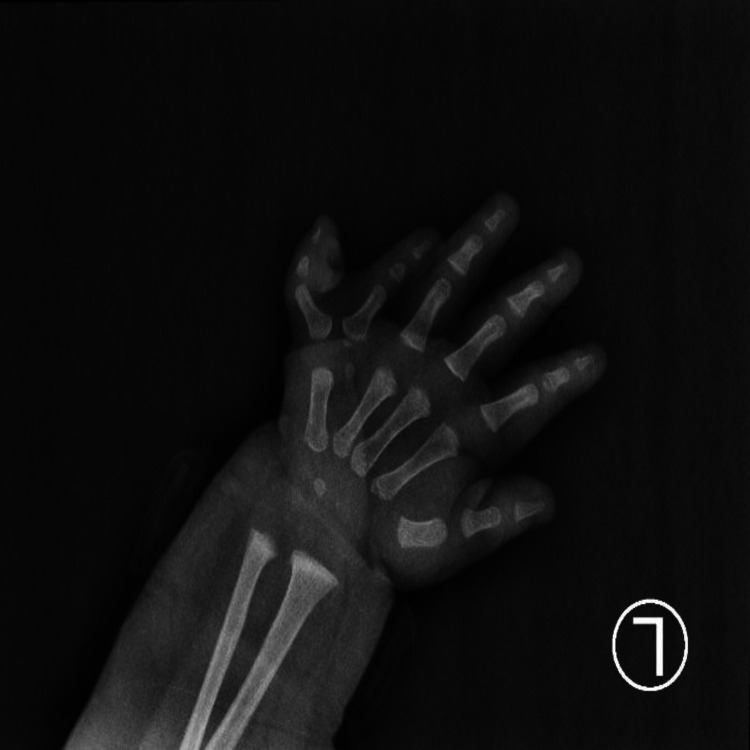
X-rays of the patient’s left hand showing postaxial polydactyly (Stelling and Turek type 2)

A radiograph of the left hand showed a postaxial polydactyly (Figure 4).

**Figure 5:**
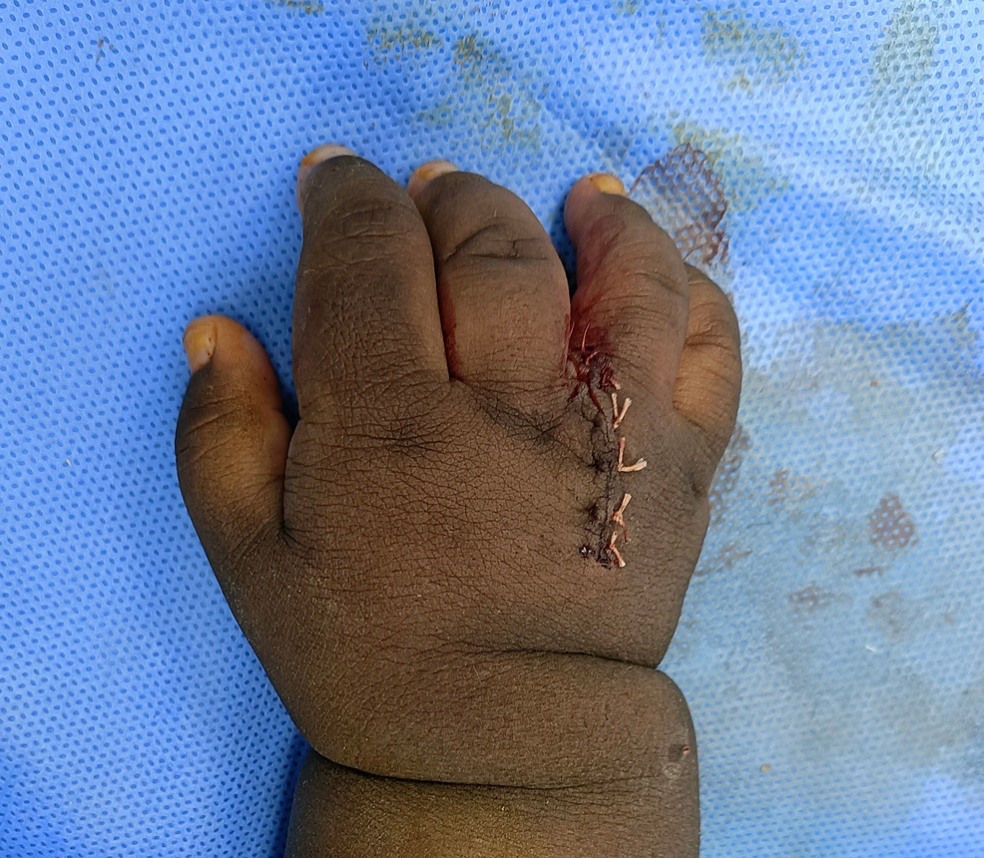
Postoperative picture of the dorsum of the right hand after resection

**Figure 6:**
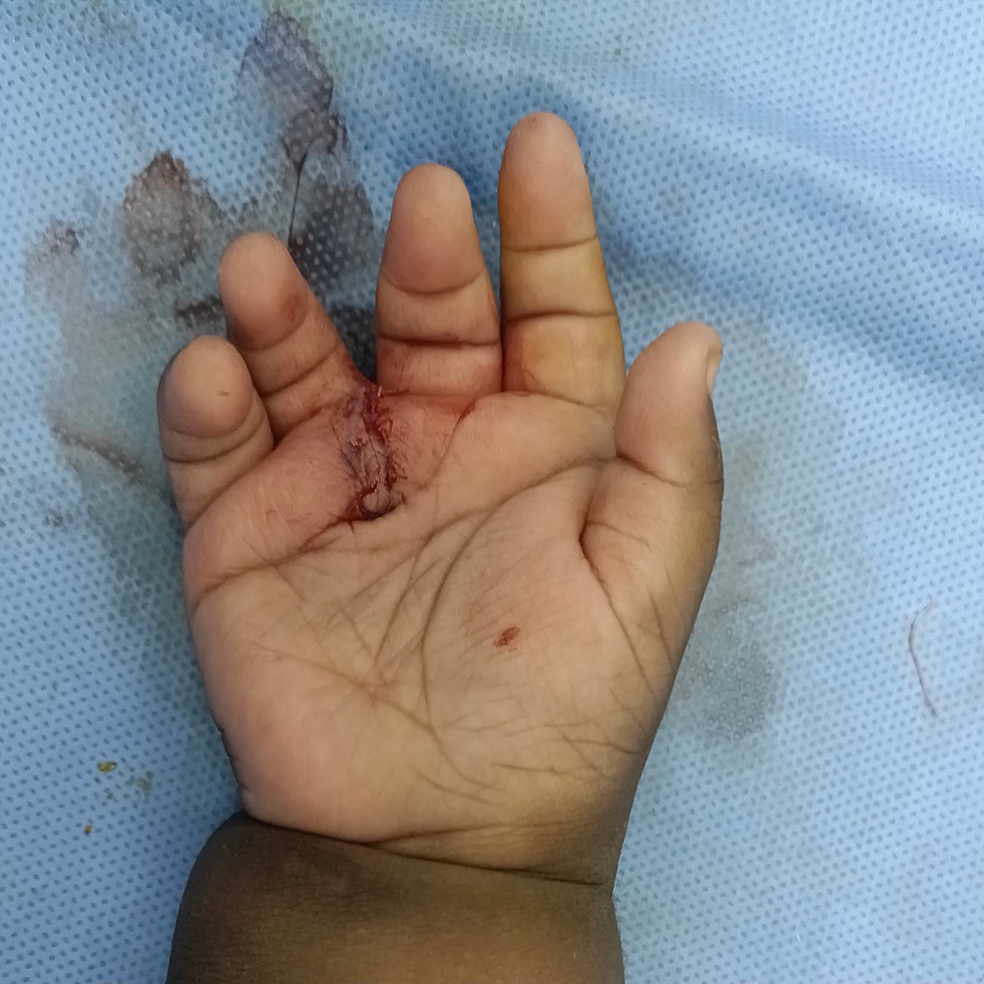
Postoperative picture of the palm of the right hand

The child was operated on under general anesthesia with oral intubation. A tourniquet was applied and the pressure was kept at 180 mmHg. A tennis racquet incision was marked on the right hand. After the incision, sharp dissection was done with tenotomy scissors. Then, transfer of the tendinous and ligamentous structures to the remaining dominant digit was done to achieve good function. After periosteal ligamental flap elevation, excision of the extra digit was done. In addition, osteotomy of the third bifid metacarpal bone was done using a small size osteotome, and repair of the joint capsule and the transverse metacarpal ligament was done using 3-0 Prolene (Ethicon Inc., Georgia, USA) (Figure 5-6).

**Figure 7:**
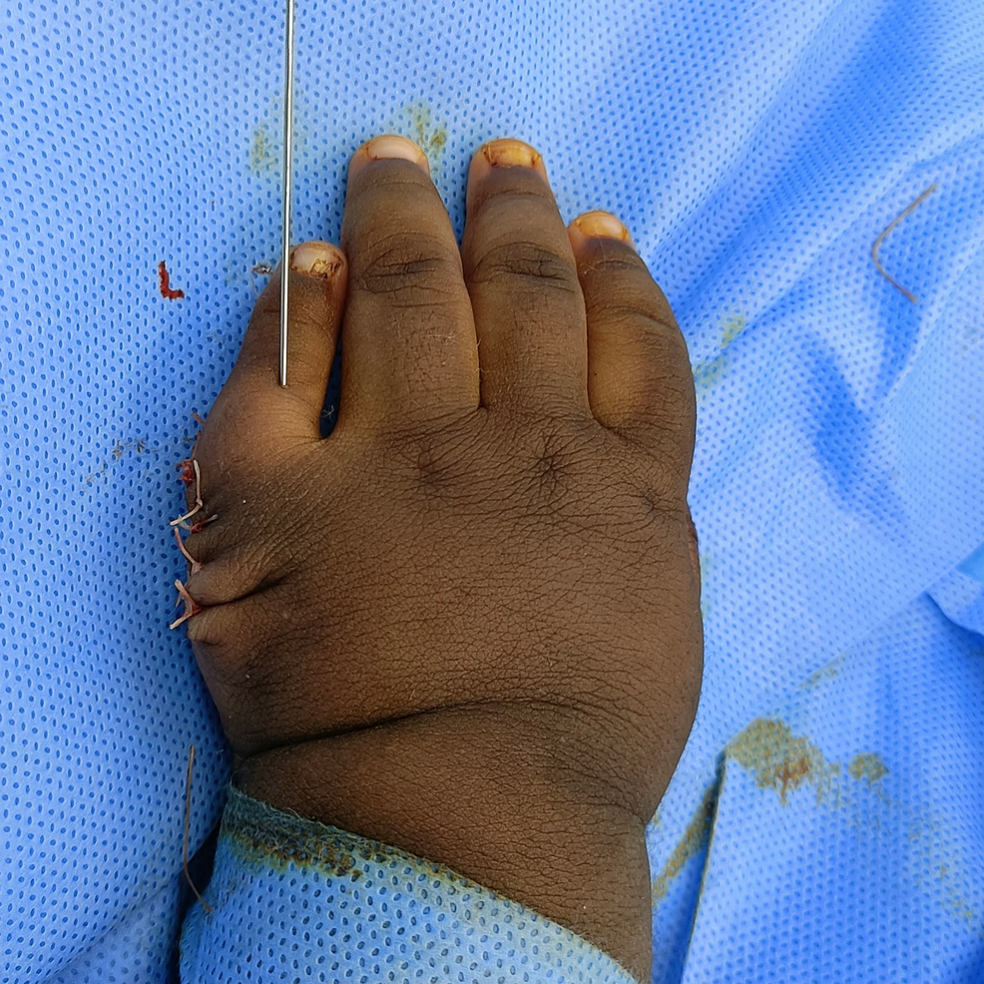
Postoperative picture of the patient’s left hand after repair with fixation wire inserted in the fifth digit

Similarly, for the left hand, excision of the extra digit was done. The little finger was realigned with a small size k-wire, 0.28 inch (0.7 mm) in diameter and 8 inches in length, with capsulorrhaphy of the metacarpophalangeal joint (Figure 7).

The wounds were closed in two layers. Then, a volar slab was applied to both hands in a functional position and was kept for two weeks.

## Discussion

**Figure 8:**
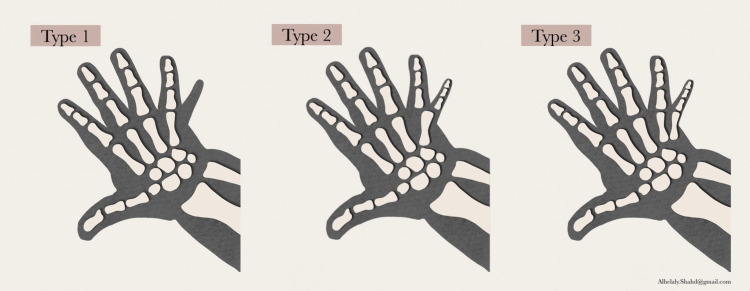
According to Stelling and Turek’s Classification, there are three types of ulnar polydactyly In type 1, there is soft tissue duplication. In type 2, there is a complete digit. In type 3, there is a complete ray including a metacarpal.

Polydactyly, the most common congenital malformation of the hand, has a wide spectrum of clinical presentations ranging from simple to complex and hence there is a paucity of any standardized treatment for it. Treatment is mainly guided by the anticipated function of the extra digit and the cosmetic deformity. Even though there are numerous techniques for the surgical management of hand polydactyly, the general principles include proper planning to achieve a straight, strong functional digit with no residual deformity. In addition, preservation of the soft tissues and excision of the accessory digit accompanied with realignment and repair of the ligaments are essential to achieve stability of the digit. To simplify the surgical management, ulnar polydactyly, which is the commonest, is further divided into three types according to the Stelling and Turek classification based on the contents of the duplicated part [8]. Type 1 is when the duplicated part contains soft tissues only, type 2 is when it contains osseous structures, and type 3 is when there is a fully developed extra digit with a metacarpal [8]. This classification is demonstrated in Figure 8.

In addition to this complexity, the type and severity of postaxial polydactyly to some extent are related to ethnicity as well. It is more common in African populations with an incidence of one in every 100 to 300 live births compared to Caucasian children in which the incidence of postaxial polydactyly is one in every 1500 to 3000 live births [2,9]. In those of African descent, postaxial polydactyly tends to be bilateral in the majority of cases and is usually isolated and autosomal dominant [9]. In other populations, it tends to be unilateral and sporadic [9]. Our case supports the common finding that ulnar polydactyly is isolated, but it is unusual in that it is unilateral ulnar polydactyly and there is no family history of note to suggest an autosomal dominant mode of inheritance. The management of postaxial polydactyly ranges from suture ligation to surgical excision depending on the contents of the extra digit [10]. Ligation is a safe, effective option when the extra digit is mainly composed of soft tissues [11]. In this case, given the nature of the duplication, the patient underwent surgical resection rather than ligation, and the postoperative outcomes were promising.

Central polydactyly is when the duplication affects the index, middle, or ring fingers. It is relatively uncommon, and, when encountered, it is most frequently associated with syndactyly or cleft hand [5,12]. Central polydactyly is mainly treated surgically. In a case series of 12 patients with central polydactyly, it was reported that patients suffered contracture or angular deformity in the operated hand [13]. Central polydactyly requires thorough, creative preoperative planning as there is limited clinical data on the most optimal surgical technique, and there are many postoperative concerns regarding cosmesis and functionality. Only very few cases of central polydactyly have been reported [13-16], and the majority if not all were associated with syndactyly, cleft hand, or a syndromic disease. To our knowledge, this is the first case report on isolated central polydactyly of the hand. This can help determine whether the postoperative complications commonly reported are mainly due to the associations that usually present with central polydactyly, and it can further inform on the most appropriate surgical approach.

In a patient with polydactyly, a comprehensive diagnostic approach is necessary to rule out any syndromic associations and to assess suitability for genetic counseling. Patients also require radiographic assessment to check for skeletal elements and bone alignment for better surgical planning. The main line of treatment is surgical resection and reconstruction taking into account the type and severity of duplication and the digits involved. This is to enhance cosmesis and improve functioning leading to a better quality of life. Surgical correction is recommended at the age of six to nine months to ensure normal anatomy before the development of fine motor skills [17]. However, it is not uncommon for surgery to be performed around the age of one year [18] and successful results have been reported in surgeries performed on adults [19]. High-quality care is essential during preoperative planning and the surgical procedure to minimize the risk of reoperation.

## Conclusions

Polydactyly is the most common congenital hand anomaly with a clinical presentation that varies widely. Central polydactyly is considered relatively very rare, and this makes its management challenging with an increased risk of contracture postoperatively. Hence, it requires long-term patient follow-up. More research is needed to inform the best surgical approach with minimal complications. In contrast to central polydactyly, postaxial polydactyly is the most frequently encountered type. Even though surgery is usually the main modality of treatment, it is controversial in ulnar polydactyly. Some surgeons prefer suture ligation to surgical resection. In this case of isolated, complex bilateral polydactyly, the patient underwent surgical correction of central and postaxial polydactyly with excellent immediate postoperative results. The patient will be followed up for any contracture and deformities to ensure good functioning and quality of life. Polydactyly can be challenging as it may be associated with other medical problems. Therefore, a thorough preoperative assessment with detailed physical examination and radiological imaging is required to avoid complications and improve functioning. Patient follow-up is essential to detect any complications at an early stage and treatment should be personalized.
